# My Commando Experiences in the Rebellion

**Published:** 1915-06-12

**Authors:** 


### MY COMMANDO EXPERIENCES IN THE REBELLION.

### By a South African Medical Officer.

The late rebellion in South Africa furnished some interesting notes, although, in comparison with the fighting in Europe, the skirmishes can hardly be called severe. Yet whether a man is wounded in a great battle or in an outpost affair, the medical interest is as great, provided the conditions of the wound are sufficient to attract attention. In the clear, healthy South African atmosphere wounds heal with surprising rapidity, and without any of those untoward complications that are so frequent in Europe, where the battlefield conditions are, from a surgical point of view, much less favourable to the wounded. Again, there have been no shell wounds among the men wounded in the rebellion fights. Big guns were only used once, so far as I am aware—at the fight against De Wet at Mushroom Valley—and there their execution was nil. On the other hand, wounds by so-called dum-dum or soft-nosed bullets, generally the Martini sporting variety, were not infrequent, and inflicted huge gashes. One of the most serious wounds I personally saw was caused by a round leaden ball fired from an elephant gun used by one of the rebels at Nooitgedacht, where the rebel commandant, Fourie, afterwards executed, made his last stand. This fight was one of the most exciting and serious during the whole rebel campaign, and the shooting was at comparatively close quarters, the first man on the side of the Government being wounded at a distance of three hundred yards, and the last at a distance of some ten yards, while two bayonet wounds were recorded. The fight began shortly before sunset on a rather dull, rainy day, and was finished an hour after dark. It took place in thickly wooded country, the rebels hiding in dongas between huge mimosa trees, and there was consequently a great deal of difficulty in tracing the wounded afterwards. The ambulance cart had remained far behind; owing to the mud and the bad condition of the veld it had been unable to proceed, and the medical officers were forced to rely on such simple equipment as their haversacks contained.

### Nature of the Wounds.

Skirmishes of this nature were fairly frequent during the campaign. The majority of them yielded a death-roll of half a dozen or so and a casualty list, so far as the wounded were concerned, of from a dozen to fifty. The great majority of the wounds were clean-cut rifle wounds, and the only complications experienced were when the bullet had in its course inflicted damage upon a viscus or a nerve. Wounds implicating a large vessel were rare; wounds of the nerves, on the other hand, by no means so, while fractures were equally common. Some interesting cases of visceral wounds were observed. One of these was a bullet wound, injuring the urethra, neck of the bladder, and the lower portion of the rectum; another was a bullet wound that entered the left flank obliquely, perforated the base of the lung, the diaphragm, and the spleen, and emerged in front two inches below the left costal margin. This wounded burgher, when carried off the field, was in an extremely critical condition, as there had been considerable internal hæmorrhage, but all that could be done for him at the moment was to dress his wound and to inject morphia to relieve his pain and distress. Notwithstanding this rough-and-ready treatment he made a good recovery, complicated by an empyema that was evacuated in hospital. The burgher wounded in the rectum and bladder is still in hospital [at the time of writing] with a recto-urinary fistula, for which a plastic operation will be necessary.

### Two Strange Cases.

Among the interesting cases in my notes are those of a picket of five men, located on an ironstone kopje where, during a sévere thunderstorm, they were struck by lightning. Three of them were killed on the spot and the other two knocked unconscious. When picked up by the relief they were found lying together in a heap, and it was at first presumed that they had been killed in some other manner, as there were no signs of the havoc usually wrought by a lightning flash—none had his clothes torn. The two unconscious men were removed to hospital, where it was found that they presented several marks of scorching on their limbs and trunk, while their watches, buttons, and other metallic instruments about their persons were twisted and fused. One recovered rapidly, suffering from little else but shock; the other was much more unfortunate, as he suffered from diplopia, external squint, paralysis of the right shoulder and entire right arm, and slight paralysis of the right leg. It is interesting to speculate on the possible lesions that accounted for this condition, but unfortunately we are much too busy with practical details to devote attention at present to the highly interesting scientific points that crop up for speculative discussion.

One of these is the treatment of the typhoid carrier. In a country where the disease is endemic, and where all the water supplies are not above suspicion, the importance of the typhoid carrier is obvious. After much cogitation the Defence Department has laid down a rule in General Orders that men who have contracted the disease on active service shall be discharged from service and exempted from peace training for at least a year. This seems the only logical manner in which to deal with these cases, since it has been proved that convalescents may communicate the disease months after their recovery. Indeed, it is impossible to lay down a definite rule when a patient who has had typhoid ceases to be a source of infection to the community; only repeated and accurate bacteriological examination can prove him to be free from all suspicion of being a carrier, and it is obviously impossible to carry out such frequent examinations on active service. Therefore, since prevention is better than cure, the attitude of the Defence Department is fully justified, although it is sometimes very difficult to make an enthusiastic burgher who has volunteered for service in German South-West Africa realise that his presence in a commando may be a source of danger to his fellow-burghers.

Bully Beef and Poison Cases.

Another interesting point that has been raised, and that is now being investigated, is the possibility of nitrite and nitrate poisoning through the consumption of "bully beef" served out as a ration on active service. This subject has received very little attention, though there is strong reason to believe that many of the cases of dysenteric diarrhœa that occurred during the last Boer War were really cases of such poisoning by nitrites due to the decomposition of the nitrates in the canned beef. Major Beveridge, of the R.A.M.C., has drawn attention to the possibilities of nitrate poisoning, but the subject is worth further investigation. When we consider that nitrate of potassium is a powerful heart depressant, and as a poison only less active than potassium chlorate, it is easy to see that where the meat has been preserved by the addition of comparatively large quantities of this salt serious symptoms may arise through the consumption of such meat, especially when it is eaten in a dry climate where the soldiers have little opportunity to drink large quantities of water, and thus, by flushing the kidneys, to get rid speedily of the excess of the salt.

Wound infection, of which we hear so much in the professional Press from the men at the European Front, has scarcely been in evidence here. One reason for its comparative infrequency is the dry atmosphere. Even when it is dust-laden it seems to be inimical to the growth of septic organisms. I believe during the last Boer War numerous investigations were made by the R.A.M.C. specialists to find out if Karroo dust harboured pathogenic organisms, but all the experiments were negative. Another reason is undoubtedly the fine stamina of the men on commando. The majority of them have led a healthy, out-of-doors existence all their lives, and their resistance to septic organisms is comparatively high. Since a thorough medical inspection of all recruits has been instituted, the physique of the men on commando has been measurably raised, and recent statistical returns of invalidity among the troops in German South-West Africa show that the percentage of sick is very low—much lower than one would have expected in a country which is malarious, and where the hardships of the campaign are undoubtedly very great.

The means of first-aid dressings in the field have generally consisted in a liberal application of iodine, followed by a simple dressing of sterile gauze, over which a pledget of wool is placed, and the whole bound tight with a bandage. This has served very well, and even in cases where the wounds have been dirtied by mud, animal excrement, and dust, the results have been very favourable. Cases of severe gangrene have only been noted where the wounds have been unduly severe, so as to cut off the blood and nerve supply to a part of the limb. In such cases amputation has been the rule, almost invariably with excellent results.

